# Supplementary figures and images for: Genetic relationships of Aspergillus fumigatus in hospital settings during COVID-19
Source: Microbiol Spectr. 2025 Apr 2;13(5):e01902-24. doi: 10.1128/spectrum.01902-24 (PMC12054129; doi:10.1128/spectrum.01902-24)

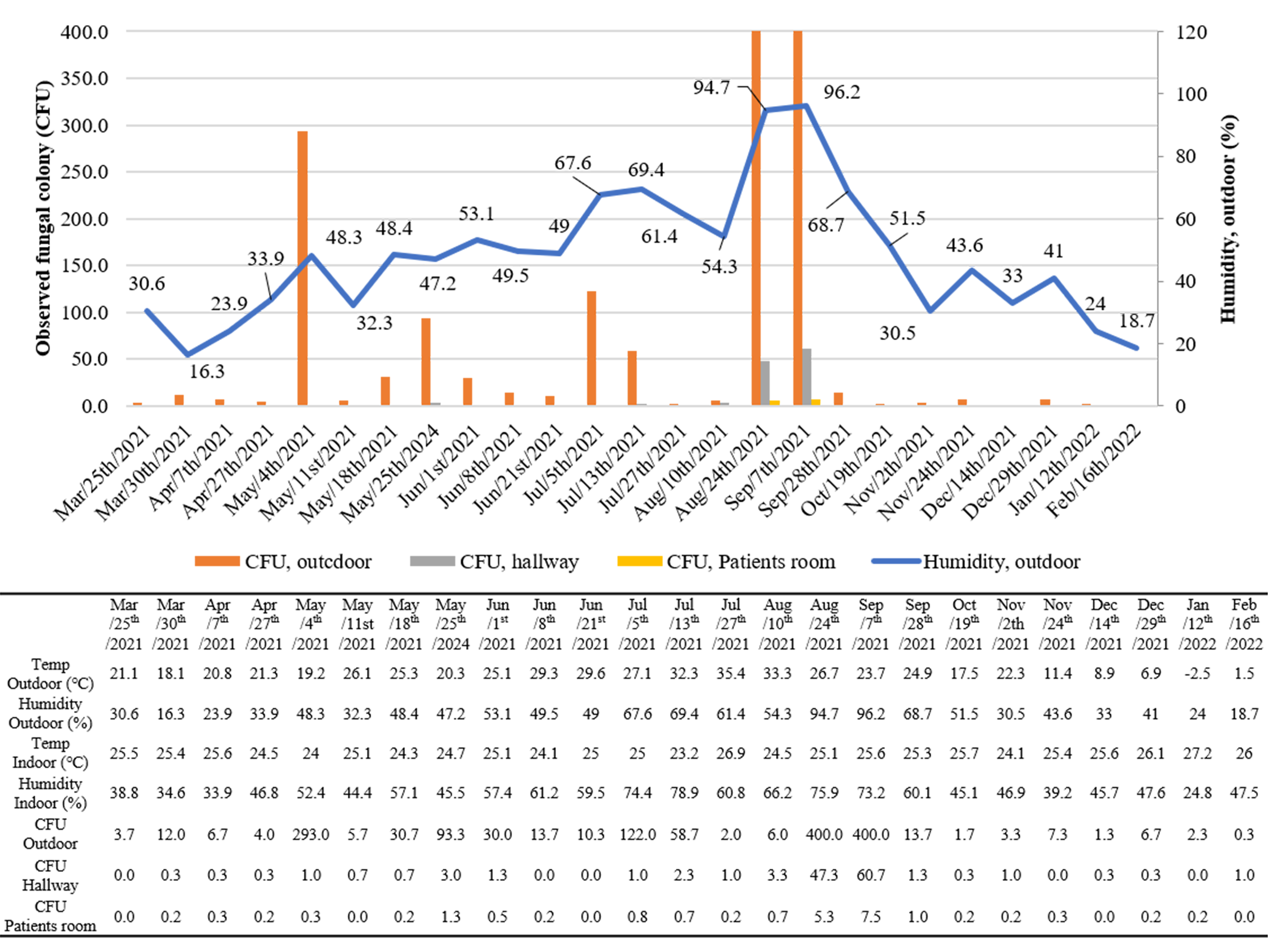

Supplement: Figure S1 — Fungal colony counts and environmental conditions at each sampling site. [file spectrum.01902-24-s0001.tif]

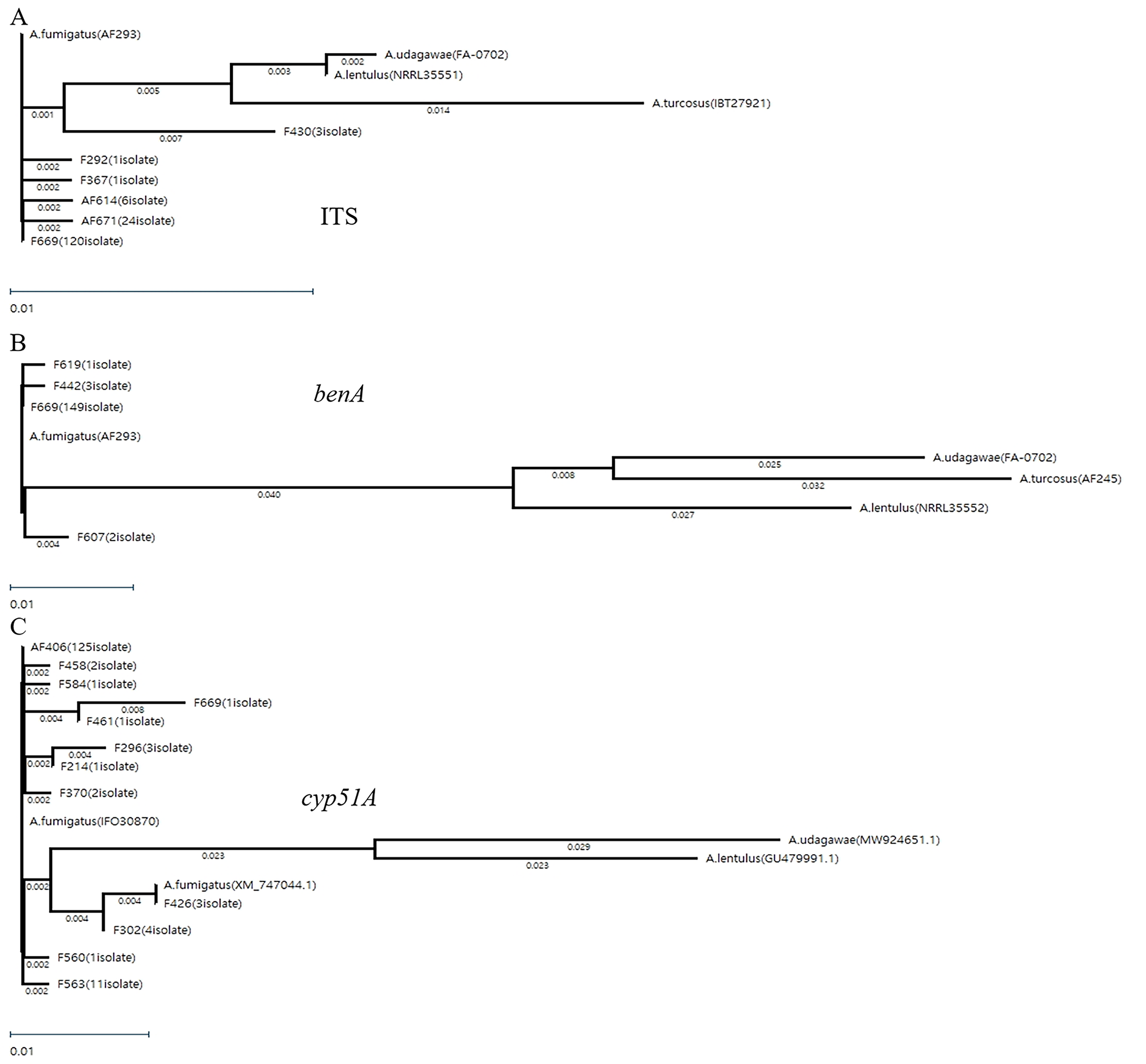

Supplement: Figure S2 — Phylogenetic tree of Aspergillus strains based on ITS, benA, and cyp51A gene sequences. [file spectrum.01902-24-s0002.tif]
