# Supplementary material for: Genetic relationships of Aspergillus fumigatus in hospital settings during COVID-19
Source: Microbiol Spectr. 2025 Apr 2;13(5):e01902-24. doi: 10.1128/spectrum.01902-24 (PMC12054129; doi:10.1128/spectrum.01902-24)
Supplement: Supplemental material — Supplemental method, tables, and figures. [file spectrum.01902-24-s0003.docx]

**Supplementary data**

Supplementary Methods……………………………………………………...………………. 2

Supplementary Table S1……….…………………………………………………...…………4

Supplementary Table S2……………………………….……………………….….…….……5

Supplementary figure legends……...…………………………………………….……….…..6

References……………………………………………………………………….….…………7

**SUPPLEMENTARY METHODS**

**Environmental Air Sampling and Culture**

Air sampling was conducted using a KAS-110 air sampler (Kemic Corp, Korea, approved by the Ministry of Environment, approval number IASM-2012-2). Samples were collected at a flow rate of 16 liters per minute for 15 minutes, followed by a 20-minute break to allow for air circulation, and this process was repeated three times at each spot. Sabouraud dextrose agar (SDA) plates were used as the collection substrate and incubated at 35°C for 4-5 days. The number of colony-forming units (CFUs) of fungi was counted every 24 hours, and the final fungal colonies in the air were sub-cultured onto new SDA plates. The fungal colony count was determined by calculating the average CFUs from the three plates collected at each sampling location. Temperature and humidity were verified using a calibrated thermohygrometer to ensure proper air collection conditions.

**Identification and Susceptibility Test**

Fungal isolates obtained from environmental monitoring samples were sub-cultured at the single colony level. Up to four colonies per plate, presumed to be identical, were selected and isolated. Clinical isolates were selected through sub-culturing single colonies from clinical specimens. To identify the species of the selected isolates, partial sequence analysis of the internal transcribed spacer (ITS) and *β-tubulin A* (*benA*) genes was performed. The primers used for each target are listed in **Table S1**, and PCR was performed under specific conditions. Only isolates confirmed as *Aspergillus fumigatus* through PCR were used in this study.

The minimum inhibitory concentrations (MICs) and minimum effective concentrations (MECs) of various antifungal agents against *A. fumigatus* isolates were determined using the broth microdilution method, following the guidelines of the Clinical and Laboratory Standards Institute (CLSI) document M38-A2 (1, 2). The tested antifungal agents included itraconazole (Sigma-Aldrich, St. Louis, MO, USA), voriconazole (Pfizer, Inc., New York, NY, USA), posaconazole, and amphotericin B powder (Sigma-Aldrich). MIC was defined as the lowest concentration of the antifungal agent that inhibited fungal growth by 50% compared to the control well, as assessed microscopically at 24 and 48 hours. MEC was defined as the lowest concentration that caused abnormal hyphal growth. Additional antifungal agents tested included caspofungin (Merck & Co., Inc., Kenilworth, NJ, USA), anidulafungin (Pfizer), and micafungin (Astellas Pharma, Inc., Tokyo, Japan). *Candida parapsilosis* ATCC 22019 was used as the control strain for each antifungal susceptibility test. Established concentration values and published reports suggested by the CLSI were used to determine the azole resistance status of *Aspergillus* species (3, 4).

Similarly, PCR for azole resistance analysis was performed using the *cyp51*A primer set from Table S1, followed by Sanger sequencing (MACROGEN Co., Seoul, Korea). Gene analysis, including gene merging, cluster analysis, and point mutation analysis, was conducted using the DNAStar Lasergene (version 15 software package, Madison, WI, USA) program. Through these phenotypic and genotypic analyses, azole-resistant isolates among the selected *A. fumigatus* strains were identified.

**TABLE S1** List of primers used for *Aspergillus fumigatus* species identification and azole resistance gene analysis

| Purpose | Target region | primer | sequence (5'-3') | PCR condition | Reference |
| --- | --- | --- | --- | --- | --- |
| Species identification | ITS | ITS-1F | CTTGGTCATTTAGAGGAAGTAA | 35 cycle  95℃ 30s  52℃ 30s  72℃ 30s | [5] |
|  |  | ITS-4R | TCCTCCGCTTATTGATATGC |  |  |
|  | *benA* | bt2a | GGTAACCAAATCGGTGCTGCTTTC | 35 cycle  95℃ 30s  54℃ 30s  72℃ 30s | [5] |
|  |  | bt2b | ACCCTCAGTGTAGTGACCCTTGGC |  |  |
| Azole  resistance  identification | *cyp51A*  genome | wcyp51A-F | TAATCGCAGCACCACTTCAG | 40 cycle  95℃ 30s  59℃ 30s  72℃ 60s | [5] |
|  |  | wcyp51A-R | CCGATCACACCAAATCCTTT |  |  |
|  |  | 401_seqF | ACGGCAATCTTGCTCAATGTTGTTT |  |  |
|  |  | 1501_seqF | ACGGATTCACTCCTCTATTCACTCTA |  |  |
|  |  | 745_seqR | AGGCACTTGCTGGCCGTTTT |  |  |
|  | *cyp51A*  promotor | AFTR-F | TAATCGCAGCACCACTTCAG | 35 cycle  95℃ 30s  59℃ 30s  72℃ 30s | [5] |
|  |  | AFTR-R | GCCTAGGACAAGGACGAATG |  |  |

ITS, internal transcribed spacer, *benA*: *β-tubulin A*, *cyp51A,* cytochrome P450 sterol 14α-demethylase, F: Forward, R: Reverse.

**TABLE S2** Composition of the primer set used for multiple locus variable-number tandem repeat analysis

|  | VNTR |  | Fluorochromes | Primer sequences (5’–3’) | Allele size  range (bp) |
| --- | --- | --- | --- | --- | --- |
| Multiplex 1 CE 1 | Asp 167 | Forward | FAM | TGAGATGGTTAACTTACGTAGCGC | 374-422 |
|  |  | Reverse |  | CGCTCCCACCGTTACCAAC |  |
|  | Asp 330 | Forward | ROX | ATCTGGTCGCGAAATTCCTCT | 151-218 |
|  |  | Reverse |  | TCTTCGGCCTTTTCATCCC |  |
|  | Asp 345 | Forward | TAMRA | TCTCCAACCCTTCGGACG | 191-246 |
|  |  | Reverse |  | GCCGGAAGAGCATGAAGACA |  |
|  | Asp 443 | Forward | FAM | AAGCTTCGTCTGGCGAAGAG | 154-280 |
|  |  | Reverse |  | GCACGTGTACGGTGTTCCTG |  |
|  | Asp 446 | Forward | HEX | CGATCATGTTTGCCTGAGGA | 197-260 |
|  |  | Reverse |  | CCGACAGCATCGAGCAACTA |  |
| Multiplex 2  CE 2 | Asp 20 | Forward | HEX | GGGAAGAGAGGAACCGATCC | 182-222 |
|  |  | Reverse |  | CGCAGTGGGCAGTTTGAAT |  |
|  | Asp 165 | Forward | TAMRA | TGATGGGCCGCAGTCG | 154-214 |
|  |  | Reverse |  | GCACCTGCTTGTCGATTCGT |  |
|  | Asp 202 | Forward | FAM | AGGATCACTGCCCTCAACCC | 200-296 |
|  |  | Reverse |  | CCGAAATCCGCGGGA |  |
|  | Asp 204bis | Forward | FAM | ATTGGGAAGAGACGGGGTAT | 134-178 |
|  |  | Reverse |  | GTCCTCACTTTTGCCTTGGT |  |
|  | Asp 252 | Forward | ROX | CAGATTGGAGACACGAAGCG | 176-224 |
|  |  | Reverse |  | ACCACGGATTGCCAAGGA |  |

MLVA: multiple locus variable-number tandem repeat analysis, bp: base pair, CE: Capillary electrophoresis

**Supplementary figure legends**

**FIG S1. Fungal colony counts and environmental conditions at each sampling site.** This graph shows the average number of fungal colonies cultured from air samples collected at various locations. Indoor and outdoor colony counts correlate with external humidity levels. CFU, colony forming units; Temp, temperature.

**FIG S2. Phylogenetic tree of *Aspergillus* strains based on ITS, *benA*, and *cyp51A* gene sequences.** This figure displays the phylogenetic tree constructed from sequence analyses of the ITS, *benA*, and *cyp51A* genes. The analysis integrates redundant sequencing data, showing that most strains align closely with the reference genes of *Aspergillus fumigatus*. No significant genetic differences were observed among *A. fumigatus* strains in all gene analyses, highlighting the potential limitations of these markers for detailed epidemiological differentiation. ITS, internal transcribed spacer; *benA*, *β-tubulin A*; *cyp51A*, cytochrome P450 14-α sterol demethylase.

**References**

1. Clinical and Laboratory Standards Institute. 2008. Reference method for broth dilution antifungal susceptibility testing of filamentous fungi: approved standard, 2nd ed. CLSI document M38-A2. CLSI, Wayne, PA.
2. Clinical and Laboratory Standards Institute. 2016. Epidemiological cutoff values for antifungal susceptibility testing. CLSI supplement M59. CLSI, Wayne, PA.
3. Espinel-Ingroff A, Turnidge J. 2016. The role of epidemiological cutoff values (ECVs/ECOFFs) in antifungal susceptibility testing and interpretation for uncommon yeasts and moulds. Rev Iberoam Micol 33:63-75.
4. Espinel-Ingroff A, Turnidge J, Alastruey-Izquierdo A, Dannaoui E, Garcia-Effron G, Guinea J, Kidd S, Pelaez T, Sanguinetti M, Meletiadis J, Botterel F, Bustamante B, Chen YC, Chakrabarti A, Chowdhary A, Chryssanthou E, Cordoba S, Gonzalez GM, Guarro J, Johnson EM, Kus JV, Lass-Florl C, Linares-Sicilia MJ, Martin-Mazuelos E, Negri CE, Pfaller MA, Tortorano AM. 2018. Posaconazole MIC Distributions for Aspergillus fumigatus Species Complex by Four Methods: Impact of cyp51A Mutations on Estimation of Epidemiological Cutoff Values. Antimicrob Agents Chemother 62.
5. Sung-Yeon Cho, Dong-Gun Lee, Won-Bok Kim, Hye-Sun Chun, Chulmin Park, Jun-Pyo Myong, Yeon-Joon Park, Jae-Ki Choi, Hyo-Jin Lee, Si-Hyun Kim, Sun Hee Park, Su-Mi Choi, Jung-Hyun Choi, and Jin-Hong Yoo, Epidemiology and Antifungal Susceptibility Profile of Aspergillus Species: Comparison between Environmental and Clinical Isolates from Patients with Hematologic Malignancies, J Clin Microbiol. 2019 Jul; 57(7): e02023-18.
