# Supplementary material for: Genetic relationships of Aspergillus fumigatus in hospital settings during COVID-19
Source: Microbiol Spectr. 2025 Apr 2;13(5):e01902-24. doi: 10.1128/spectrum.01902-24 (PMC12054129; doi:10.1128/spectrum.01902-24)
Supplement: Supplemental file — All results of multiple locus variable-number tandem repeat sequence types. [file spectrum.01902-24-s0004.pdf]

strain	ST	Asp167	Asp330	Asp345	Asp443	Asp446	Asp20	Asp165	Asp202	Asp204bis	Asp252	GT	location	collection	date	azole resistance	specimen	point mutation	Itraconazole	Voriconazole	Posaconazole
AF406	58	7	2	3	7	1	5	3	5	3	3	7-2-3-7-1-5-3-5-3-3	KOREA	Environmental	2021-03-25	WT	outdoor	-	0.25	0.06	0.25
AF431	64	7	4	3	3	3	5	2	2	2	3	7-4-3-3-3-5-2-2-2-3	KOREA	Environmental	2021-04-27	WT	outdoor	-	0.5	0.06	0.25
AF433	13	6	2	2	7	1	6	2	4	1	3	6-2-2-7-1-6-2-4-1-3	KOREA	Environmental	2021-04-27	WT	outdoor	-	0.5	0.06	0.5
AF434	13	6	2	2	7	1	6	2	4	1	3	6-2-2-7-1-6-2-4-1-3	KOREA	Environmental	2021-04-27	WT	outdoor	-	0.5	0.06	0.5
AF444	93	8	5	3	5	3	5	4	10	3	3	8-5-3-5-3-5-4-10-3-3	KOREA	Environmental	2021-05-04	WT	indoor-entrance	-	0.25	0.06	0.25
AF463	67	7	4	3	5	2	5	2	8	3	3	7-4-3-5-2-5-2-8-3-3	KOREA	Environmental	2021-05-25	WT	outdoor	-	0.25	0.06	0.125
AF479	84	8	2	3	6	1	5	2	8	3	3	8-2-3-6-1-5-2-8-3-3	KOREA	Environmental	2021-06-01	WT	outdoor	-	0.25	0.06	0.25
AF480	83	8	2	3	6	1	5	2	10	1	3	8-2-3-6-1-5-2-10-1-3	KOREA	Environmental	2021-06-01	WT	outdoor	-	0.25	0.06	0.5
AF500	55	6	9	3	7	1	5	2	10	1	3	6-9-3-7-1-5-2-10-1-3	KOREA	Environmental	2021-07-05	WT	outdoor	-	0.5	0.06	0.25
AF501	54	6	9	3	7	1	5	1	6	2	3	6-9-3-7-1-5-1-6-2-3	KOREA	Environmental	2021-07-05	WT	outdoor	-	0.25	0.06	0.25
AF513	7	5	5	3	3	3	4	2	6	4	3	5-5-3-3-3-4-2-6-4-3	KOREA	Environmental	2021-07-13	WT	outdoor	-	0.5	0.06	0.25
AF516	65	7	4	3	4	3	4	3	5	4	3	7-4-3-4-3-4-3-5-4-3	KOREA	Environmental	2021-07-13	WT	outdoor	-	0.25	0.06	0.5
AF526	108	10	2	3	6	3	5	2	10	4	0	10-2-3-6-3-5-2-10-4-0	KOREA	Environmental	2021-07-24	WT	outdoor	-	0.5	0.06	0.5
AF575	95	9	2	3	4	1	5	3	4	3	3	9-2-3-4-1-5-3-4-3-3	KOREA	Environmental	2021-09-07	WT	outdoor	-	0.25	0.06	0.25
AF577	9	5	5	3	5	3	6	2	8	4	3	5-5-3-5-3-6-2-8-4-3	KOREA	Environmental	2021-09-07	WT	outdoor	-	0.5	0.06	0.25
AF579	81	8	2	3	5	1	4	2	11	3	3	8-2-3-5-1-4-2-11-3-3	KOREA	Environmental	2021-09-07	WT	outdoor	-	0.25	0.06	0.5
AF614	14	6	2	3	5	1	4	2	2	3	3	6-2-3-5-1-4-2-2-3-3	KOREA	Environmental	2021-11-02	WT	outdoor	-	0.25	0.06	0.25
AF615	69	7	4	3	5	3	4	2	2	2	3	7-4-3-5-3-4-2-2-2-3	KOREA	Environmental	2021-11-02	WT	outdoor	-	0.25	0.06	0.25
AF617	64	7	4	3	3	3	5	2	2	2	3	7-4-3-3-3-5-2-2-2-3	KOREA	Environmental	2021-11-02	WT	indoor-entrance	-	0.25	0.5	0.06
AF623	68	7	4	3	5	3	4	2	11	4	3	7-4-3-5-3-4-2-11-4-3	KOREA	Environmental	2021-11-24	WT	outdoor	-	0.25	0.25	0.06
AF624	88	8	3	3	5	2	4	2	6	3	3	8-3-3-5-2-4-2-6-3-3	KOREA	Environmental	2021-11-24	WT	outdoor	N248K	0.25	0.25	0.06
AF625	88	8	3	3	5	2	4	2	6	3	3	8-3-3-5-2-4-2-6-3-3	KOREA	Environmental	2021-11-24	WT	outdoor	N248K	0.25	0.25	0.06
AF626	40	6	5	3	5	3	5	2	7	1	3	6-5-3-5-3-5-2-7-1-3	KOREA	Environmental	2021-11-24	WT	outdoor	-	0.25	1	0.06
AF628	64	7	4	3	3	3	5	2	2	2	3	7-4-3-3-3-5-2-2-2-3	KOREA	Environmental	2021-11-24	WT	outdoor	-	0.25	0.5	0.06
AF630	64	7	4	3	3	3	5	2	2	2	3	7-4-3-3-3-5-2-2-2-3	KOREA	Environmental	2021-11-24	WT	outdoor	-	0.25	0.5	0.06
AF631	119	10	4	3	5	3	4	2	6	2	3	10-4-3-5-3-4-2-6-2-3	KOREA	Environmental	2021-11-24	WT	outdoor	-	0.25	0.5	0.06
AF632	12	6	2	1	4	1	4	2	5	4	3	6-2-1-4-1-4-2-5-4-3	KOREA	Environmental	2021-11-24	WT	outdoor	F46Y, M172V, E427K	0.5	1	0.06
AF633	46	6	5	3	7	2	5	2	6	2	3	6-5-3-7-2-5-2-6-2-3	KOREA	Environmental	2021-11-24	WT	outdoor	-	0.25	0.5	0.06
AF634	35	6	5	3	2	2	5	2	6	2	3	6-5-3-2-2-5-2-6-2-3	KOREA	Environmental	2021-11-24	WT	outdoor	-	0.5	0.5	0.06
AF636	80	7	7	3	5	3	4	3	12	3	3	7-7-3-5-3-4-3-12-3-3	KOREA	Environmental	2021-11-24	WT	indoor-patient's room	-	0.25	0.5	0.06
AF639	93	8	5	3	5	3	5	4	10	3	3	8-5-3-5-3-5-4-10-3-3	KOREA	Environmental	2021-12-14	WT	outdoor	-	0.25	0.25	0.06
AF641	127	10	7	3	3	3	5	3	11	4	3	10-7-3-3-3-5-3-11-4-3	KOREA	Environmental	2021-12-14	WT	outdoor	-	0.25	0.5	0.06
F212	25	6	3	3	5	3	5	2	7	3	4	6-3-3-5-3-5-2-7-3-4	KOREA	Non-CAPA	2020-07-23	WT	Bronchial washing fluid	-	0.5	1	0.25
F213	25	6	3	3	5	3	5	2	7	3	4	6-3-3-5-3-5-2-7-3-4	KOREA	Non-CAPA	2020-07-24	WT	Sputum	-	0.5	0.125	0.25
F214	102	10	2	3	5	1	4	2	4	4	2	10-2-3-5-1-4-2-4-4-2	KOREA	Non-CAPA	2020-07-24	TR34	Sputum(Trap)	L98H	4	4	1
F215	78	7	7	3	3	3	4	3	10	3	3	7-7-3-3-3-4-3-10-3-3	KOREA	Non-CAPA	2020-07-24	WT	Sputum(Trap)	-	0.5	0.125	0.25
F217	22	6	3	3	5	2	4	2	6	4	3	6-3-3-5-2-4-2-6-4-3	KOREA	Non-CAPA	2020-08-03	WT	Sputum	-	0.25	0.25	0.125
F218	39	6	5	3	5	3	5	2	6	3	3	6-5-3-5-3-5-2-6-3-3	KOREA	Non-CAPA	2020-08-08	WT	Sputum	-	0.25	0.5	0.25
F219	45	6	5	3	7	1	6	3	6	2	3	6-5-3-7-1-6-3-6-2-3	KOREA	Non-CAPA	2020-08-08	WT	Sputum	-	0.25	0.5	0.25
F228	86	8	2	4	8	2	4	2	6	3	3	8-2-4-8-2-4-2-6-3-3	KOREA	Non-CAPA	2020-08-12	WT	Sputum	N248K	0.25	0.25	0.125
F229	89	8	3	3	5	3	5	2	10	3	3	8-3-3-5-3-5-2-10-3-3	KOREA	Non-CAPA	2020-08-19	WT	Bronchial washing fluid	N248K	0.25	0.25	0.125
F230	115	10	4	3	4	3	4	3	5	3	3	10-4-3-4-3-4-3-5-3-3	KOREA	Non-CAPA	2020-08-26	WT	Sputum	-	0.5	0.25	0.25
F234	27	6	3	3	7	2	6	2	7	3	3	6-3-3-7-2-6-2-7-3-3	KOREA	CAPA	2020-08-23	WT	Sputum(Trap)	-	0.25	0.25	0.125
F237	23	6	3	3	5	2	4	6	6	3	3	6-3-3-5-2-4-6-6-3-3	KOREA	Non-CAPA	2020-08-31	WT	Sputum	F46Y, M172V,N248T, D255E, E427K	0.5	1	0.25
F240	59	7	2	4	7	1	5	2	7	1	3	7-2-4-7-1-5-2-7-1-3	KOREA	Non-CAPA	2020-08-31	WT	Ascites	-	0.5	0.25	0.25
F253	125	10	7	3	3	1	5	3	5	2	3	10-7-3-3-1-5-3-5-2-3	KOREA	Non-CAPA	2020-10-05	WT	Sputum	-	0.5	0.25	0.25
F256	32	6	4	3	5	3	4	3	6	2	3	6-4-3-5-3-4-3-6-2-3	KOREA	Non-CAPA	2020-10-05	WT	Bronchial washing fluid	-	0.5	0.5	0.125
F271	47	6	5	4	7	1	5	4	6	3	3	6-5-4-7-1-5-4-6-3-3	KOREA	Non-CAPA	2020-10-21	WT	Sputum	N248K	0.25	0.25	0.125
F273	16	6	2	3	7	1	5	2	6	1	3	6-2-3-7-1-5-2-6-1-3	KOREA	Non-CAPA	2020-10-22	WT	Sputum	-	0.25	0.25	0.125
F275	60	7	3	3	3	3	4	3	8	2	3	7-3-3-3-3-4-3-8-2-3	KOREA	Non-CAPA	2020-11-03	WT	Bronchial washing fluid	-	0.25	0.25	0.125
F282	125	10	7	3	3	1	5	3	5	2	3	10-7-3-3-1-5-3-5-2-3	KOREA	Non-CAPA	2020-11-19	WT	Sputum	-	0.06	0.06	0.25
F283	107	10	2	3	5	3	4	7	11	5	3	10-2-3-5-3-4-7-11-5-3	KOREA	Non-CAPA	2020-11-19	WT	Sputum(Trap)	-	0.5	0.5	0.5
F286	5	5	3	3	7	1	4	2	9	2	3	5-3-3-7-1-4-2-9-2-3	KOREA	Non-CAPA	2020-11-24	WT	Bronchial washing fluid	N248K	0.5	0.25	0.5
F287	60	7	3	3	3	3	4	3	8	2	3	7-3-3-3-3-4-3-8-2-3	KOREA	Non-CAPA	2020-11-25	WT	Sputum	-	0.5	0.5	0.5
F288	96	9	2	3	5	2	4	2	10	5	3	9-2-3-5-2-4-2-10-5-3	KOREA	CAPA	2020-11-23	WT	Sputum(Trap)	-	0.5	0.25	0.5
F291	92	8	5	3	3	3	5	2	8	3	3	8-5-3-3-3-5-2-8-3-3	KOREA	Non-CAPA	2020-12-07	WT	Sputum	-	0.06	0.06	0.25

F292	97	9	3	3	4	3	4	6	4	4	4	9-3-3-4-3-4-6-4-4-4	KOREA	Non-CAPA	2020-12-02	WT	Bronchial washing fluid	F46Y, M172V, N248T, D255E, E427K	0.25	0.06	0.25
F296	128	11	2	5	5	1	4	3	12	4	3	11-2-5-5-1-4-3-12-4-3	KOREA	Non-CAPA	2020-12-18	TR34	Sputum	L98H, S297T, F495I	64≤	1	2
F302	39	6	5	3	5	3	5	2	6	3	3	6-5-3-5-3-5-2-6-3-3	KOREA	Non-CAPA	2021-01-02	WT	Sputum	F46Y, M172V, E427K	0.25	0.06	0.25
F305	29	6	3	4	7	1	4	2	6	3	3	6-3-4-7-1-4-2-6-3-3	KOREA	Non-CAPA	2021-01-01	WT	Sputum(Trap)	N248K	0.125	0.06	0.25
F312	111	10	2	5	3	3	4	2	6	4	3	10-2-5-3-3-4-2-6-4-3	KOREA	CAPA	2021-01-08	WT	Sputum(Trap)	-	0.25	0.5	1
F313	53	6	7	3	7	1	5	2	7	2	3	6-7-3-7-1-5-2-7-2-3	KOREA	Non-CAPA	2021-01-07	WT	Bronchial washing fluid	-	0.5	0.125	0.25
F316	120	10	4	4	5	3	4	3	8	3	3	10-4-4-5-3-4-3-8-3-3	KOREA	Non-CAPA	2021-01-11	WT	Bronchial washing fluid	-	0.25	0.06	0.25
F317	33	6	4	3	5	3	5	2	6	3	3	6-4-3-5-3-5-2-6-3-3	KOREA	Non-CAPA	2021-01-14	WT	Sputum(Trap)	-	0.5	0.125	0.25
F323	31	6	4	3	5	3	4	3	11	4	3	6-4-3-5-3-4-3-11-4-3	KOREA	Non-CAPA	2021-01-25	WT	Sputum	-	0.25	0.25	0.125
F325	52	6	7	3	7	1	5	1	4	2	3	6-7-3-7-1-5-1-4-2-3	KOREA	Non-CAPA	2021-02-05	WT	Sputum	-	0.25	0.25	0.25
F327	104	10	2	3	5	2	4	6	11	3	3	10-2-3-5-2-4-6-11-3-3	KOREA	Non-CAPA	2021-02-08	WT	Sputum(Trap)	-	0.25	0.25	0.125
F330	51	6	7	3	5	3	4	2	6	3	3	6-7-3-5-3-4-2-6-3-3	KOREA	Non-CAPA	2021-02-15	WT	Sputum	N248K	0.25	0.25	0.125
F332	63	7	3	3	7	1	4	2	10	3	3	7-3-3-7-1-4-2-10-3-3	KOREA	Non-CAPA	2021-02-25	WT	Bronchial washing fluid	-	0.25	0.25	0.125
F333	91	8	4	4	5	3	5	3	7	4	3	8-4-4-5-3-5-3-7-4-3	KOREA	Non-CAPA	2021-03-09	WT	Bile	-	0.25	0.125	0.125
F334	70	7	4	3	5	3	4	3	11	4	3	7-4-3-5-3-4-3-11-4-3	KOREA	Non-CAPA	2021-03-14	WT	Sputum	-	0.25	0.125	0.06
F342	16	6	2	3	7	1	5	2	6	1	3	6-2-3-7-1-5-2-6-1-3	KOREA	Non-CAPA	2021-03-16	WT	Ear discharge	-	0.25	0.125	0.06
F348	51	6	7	3	5	3	4	2	6	3	3	6-7-3-5-3-4-2-6-3-3	KOREA	Non-CAPA	2021-03-30	WT	Sputum	N248K	0.125	0.125	0.125
F361	116	10	4	3	5	2	4	4	5	3	3	10-4-3-5-2-4-4-5-3-3	KOREA	Non-CAPA	2021-04-16	WT	Ear discharge	-	0.5	0.25	0.06
F366	85	8	2	3	6	3	4	3	4	4	3	8-2-3-6-3-4-3-4-4-3	KOREA	Non-CAPA	2021-04-28	WT	Bronchial washing fluid	-	0.5	0.25	0.125
F367	10	5	6	3	5	3	4	4	2	4	3	5-6-3-5-3-4-4-2-4-3	KOREA	Non-CAPA	2021-04-29	WT	Bronchial washing fluid	-	0.5	0.125	0.125
F368	71	7	4	3	5	3	5	4	12	3	3	7-4-3-5-3-5-4-12-3-3	KOREA	Non-CAPA	2021-04-09	WT	Tissue	-	0.5	0.25	0.125
F370	103	10	2	3	5	2	4	2	11	5	3	10-2-3-5-2-4-2-11-5-3	KOREA	Non-CAPA	2021-04-29	WT	Sputum	M39I	0.5	0.25	0.125
F371	6	5	4	3	5	3	4	2	8	6	3	5-4-3-5-3-4-2-8-6-3	KOREA	Non-CAPA	2021-04-30	WT	Bone	-	0.125	0.125	0.06
F375	66	7	4	3	4	3	5	2	10	3	3	7-4-3-4-3-5-2-10-3-3	KOREA	CAPA	2021-05-11	WT	Sputum	-	0.25	0.25	0.125
F376	17	6	2	3	7	1	5	2	6	3	3	6-2-3-7-1-5-2-6-3-3	KOREA	Non-CAPA	2021-05-10	WT	Sputum(Trap)	-	0.125	0.125	0.125
F377	103	10	2	3	5	2	4	2	11	5	3	10-2-3-5-2-4-2-11-5-3	KOREA	Non-CAPA	2021-05-10	WT	Pus	M39I	0.25	0.25	0.125
F384	110	10	2	4	5	2	4	2	8	3	4	10-2-4-5-2-4-2-8-3-4	KOREA	Non-CAPA	2021-05-09	WT	Sputum	-	0.5	0.5	0.125
F389	122	10	5	3	5	3	6	3	6	2	3	10-5-3-5-3-6-3-6-2-3	KOREA	Non-CAPA	2021-05-20	WT	Sputum(Trap)	-	0.25	0.125	0.06
F394	77	7	7	3	3	3	4	2	10	4	3	7-7-3-3-3-4-2-10-4-3	KOREA	Non-CAPA	2021-05-25	WT	Sputum	-	0.25	0.125	0.06
F398	36	6	5	3	4	2	5	3	5	3	3	6-5-3-4-2-5-3-5-3-3	KOREA	Non-CAPA	2021-05-31	WT	Tissue	-	0.125	0.25	0.12
F407	41	6	5	3	5	3	5	3	6	3	3	6-5-3-5-3-5-3-6-3-3	KOREA	Non-CAPA	2021-05-05	WT	Pleural fluid	-	0.125	0.125	0.06
F413	93	8	5	3	5	3	5	4	10	3	3	8-5-3-5-3-5-4-10-3-3	KOREA	Non-CAPA	2021-06-23	WT	Bronchial washing fluid	-	0.25	0.25	0.06
F420	126	10	7	3	3	2	4	3	5	2	3	10-7-3-3-2-4-3-5-2-3	KOREA	Non-CAPA	2021-07-08	WT	Sputum	-	0.25	0.25	0.125
F421	34	6	4	3	7	1	5	2	6	4	3	6-4-3-7-1-5-2-6-4-3	KOREA	Non-CAPA	2021-07-01	WT	Bronchial washing fluid	-	0.25	0.25	0.125
F426	48	6	6	3	5	1	4	2	6	5	3	6-6-3-5-1-4-2-6-5-3	KOREA	Non-CAPA	2021-07-06	WT	Bronchial washing fluid	F46Y, M172V, N248K, D255E, E427K	0.25	0.5	0.06
F427	20	6	2	4	5	3	5	5	8	3	3	6-2-4-5-3-5-5-8-3-3	KOREA	Non-CAPA	2021-07-12	WT	Sputum	-	0.125	0.25	0.06
F430	117	10	4	3	5	2	5	4	11	3	3	10-4-3-5-2-5-4-11-3-3	KOREA	Non-CAPA	2021-07-15	TR34	Bronchial washing fluid	L98H, S297T, F495I	64	1	1
F439	98	9	3	3	5	3	4	3	10	4	3	9-3-3-5-3-4-3-10-4-3	KOREA	Non-CAPA	2021-07-19	WT	Sputum(Trap)	-	0.5	0.5	0.125
F440	118	10	4	3	5	3	4	2	10	3	3	10-4-3-5-3-4-2-10-3-3	KOREA	Non-CAPA	2021-07-21	WT	Bronchial washing fluid	-	0.125	0.06	0.06
F442	57	7	2	2	3	1	3	3	9	1	4	7-2-2-3-1-3-3-9-1-4	KOREA	CAPA	2021-07-29	WT	Sputum(Trap)	F46Y, M172V, E427K	0.25	0.5	0.06
F456	125	10	7	3	3	1	5	3	5	2	3	10-7-3-3-1-5-3-5-2-3	KOREA	Non-CAPA	2021-08-11	WT	Sputum	-	0.25	0.25	0.06
F458	19	6	2	3	7	1	6	2	7	1	3	6-2-3-7-1-6-2-7-1-3	KOREA	Non-CAPA	2021-08-14	WT	Eye discharge	A9T	0.25	0.25	0.06
F461	123	10	6	3	7	3	4	2	7	3	3	10-6-3-7-3-4-2-7-3-3	KOREA	Non-CAPA	2021-08-16	TR46	Sputum	Y121F, T289A	1	64	0.5
F466	79	7	7	3	5	2	4	3	6	3	3	7-7-3-5-2-4-3-6-3-3	KOREA	Non-CAPA	2021-08-21	WT	Sputum	-	0.25	0.25	0.06
F467	8	5	5	3	4	3	5	2	10	3	3	5-5-3-4-3-5-2-10-3-3	KOREA	Non-CAPA	2021-08-21	WT	Sputum(Trap)	N248K	0.25	0.25	0.06
F468	125	10	7	3	3	1	5	3	5	2	3	10-7-3-3-1-5-3-5-2-3	KOREA	Non-CAPA	2021-08-22	WT	Sputum	-	0.5	0.25	0.125
F478	70	7	4	3	5	3	4	3	11	4	3	7-4-3-5-3-4-3-11-4-3	KOREA	Non-CAPA	2021-09-02	WT	Sputum	-	0.5	0.25	0.06
F485	3	5	2	3	7	1	6	2	6	4	3	5-2-3-7-1-6-2-6-4-3	KOREA	Non-CAPA	2021-09-07	WT	Sputum	-	0.5	0.25	0.125
F486	37	6	5	3	5	3	4	2	5	3	3	6-5-3-5-3-4-2-5-3-3	KOREA	Non-CAPA	2021-09-08	WT	Bronchial washing fluid	-	0.5	0.25	0.125
F491	18	6	2	3	7	1	6	2	6	4	3	6-2-3-7-1-6-2-6-4-3	KOREA	Non-CAPA	2021-09-22	WT	Sputum	-	0.5	0.25	0.125
F492	38	6	5	3	5	3	4	2	6	3	3	6-5-3-5-3-4-2-6-3-3	KOREA	Non-CAPA	2021-09-13	WT	Bronchial washing fluid	-	0.5	0.5	0.125
F493	41	6	5	3	5	3	5	3	6	3	3	6-5-3-5-3-5-3-6-3-3	KOREA	Non-CAPA	2021-09-13	WT	Bronchial washing fluid	-	0.5	0.5	0.125
F494	74	7	5	3	5	3	4	2	2	3	3	7-5-3-5-3-4-2-2-3-3	KOREA	Non-CAPA	2021-09-18	WT	Sputum	-	0.5	0.25	0.125
F501	124	10	7	3	3	1	4	2	7	4	4	10-7-3-3-1-4-2-7-4-4	KOREA	Non-CAPA	2021-09-15	none	Sputum	-	2	0.5	0.25
F513	62	7	3	3	4	3	6	2	8	4	3	7-3-3-4-3-6-2-8-4-3	KOREA	CAPA	2021-10-04	WT	Sputum	-	0.5	0.25	0.125
F516	26	6	3	3	7	2	6	2	6	3	3	6-3-3-7-2-6-2-6-3-3	KOREA	CAPA	2021-10-04	WT	Sputum	-	0.5	1	0.25
F517	24	6	3	3	5	2	5	2	6	3	3	6-3-3-5-2-5-2-6-3-3	KOREA	Non-CAPA	2021-10-08	WT	Sputum(Trap)	-	0.5	0.25	0.125

F520	99	9	3	3	5	3	4	3	9	4	2	9-3-3-5-3-4-3-9-4-2	KOREA	Non-CAPA	2021-10-15	WT	Sputum	-	0.5	0.5	0.125
F525	50	6	7	3	3	1	5	2	2	2	3	6-7-3-3-1-5-2-2-2-3	KOREA	Non-CAPA	2021-10-24	WT	Sputum	-	0.5	0.25	0.125
F526	90	8	3	3	7	1	7	3	10	3	3	8-3-3-7-1-7-3-10-3-3	KOREA	Non-CAPA	2021-10-25	WT	Sputum	-	0.5	0.25	0.125
F527	4	5	2	3	7	1	6	2	7	1	3	5-2-3-7-1-6-2-7-1-3	KOREA	Non-CAPA	2021-10-26	WT	Bronchial washing fluid	A9T	0.5	0.5	0.125
F542	72	7	5	3	4	2	4	3	10	3	3	7-5-3-4-2-4-3-10-3-3	KOREA	Non-CAPA	2021-11-01	WT	Bronchial washing fluid	-	0.5	0.125	0.125
F545	101	10	2	3	5	1	4	2	11	3	3	10-2-3-5-1-4-2-11-3-3	KOREA	CAPA	2021-11-04	WT	Sputum	-	0.5	0.25	0.125
F557	38	6	5	3	5	3	4	2	6	3	3	6-5-3-5-3-4-2-6-3-3	KOREA	CAPA	2021-11-17	WT	Sputum	-	0.25	0.125	0.06
F560	73	7	5	3	4	3	4	2	8	3	3	7-5-3-4-3-4-2-8-3-3	KOREA	Non-CAPA	2021-11-17	WT	Sputum	S335H	0.5	0.25	0.125
F563	30	6	3	4	7	1	4	3	6	3	3	6-3-4-7-1-4-3-6-3-3	KOREA	Non-CAPA	2021-11-09	WT	Sputum(Trap)	N248K	0.5	0.25	0.125
F567	44	6	5	3	7	1	5	3	6	2	3	6-5-3-7-1-5-3-6-2-3	KOREA	Non-CAPA	2021-11-24	WT	Bronchial washing fluid	-	0.5	1	0.125
F568	83	8	2	3	6	1	5	2	10	1	3	8-2-3-6-1-5-2-10-1-3	KOREA	Non-CAPA	2021-12-02	WT	Sputum(Trap)	-	0.5	0.125	0.06
F573	56	7	2	2	3	1	3	3	10	1	4	7-2-2-3-1-3-3-10-1-4	KOREA	CAPA	2021-12-09	WT	Sputum(Trap)	F46Y, M172V, E427K	0.5	0.5	0.125
F584	76	7	5	4	5	2	4	2	7	4	4	7-5-4-5-2-4-2-7-4-4	KOREA	Non-CAPA	2021-12-21	WT	Sputum(Trap)	M220T			
F590	21	6	3	3	5	2	4	2	6	3	3	6-3-3-5-2-4-2-6-3-3	KOREA	CAPA	2021-12-23	WT	Sputum(Trap)	-	0.5	0.25	0.125
F592	87	8	3	3	4	2	6	3	10	6	3	8-3-3-4-2-6-3-10-6-3	KOREA	Non-CAPA	2021-12-31	WT	Sputum	-	0.5	0.125	0.125
F598	28	6	3	3	7	3	4	2	6	2	3	6-3-3-7-3-4-2-6-2-3	KOREA	Non-CAPA	2022-01-17	WT	Tissue (Lung)	-	0.5	0.125	0.06
F602	100	10	2	2	5	3	4	6	11	3	3	10-2-2-5-3-4-6-11-3-3	KOREA	Non-CAPA	2022-01-26	WT	Bronchial washing fluid	-	0.5	0.5	0.06
F607	87	8	3	3	4	2	6	3	10	6	3	8-3-3-4-2-6-3-10-6-3	KOREA	Non-CAPA	2022-01-24	WT	Sputum	-	0.5	0.5	0.125
F608	94	8	5	4	5	3	4	4	9	4	3	8-5-4-5-3-4-4-9-4-3	KOREA	Non-CAPA	2022-02-09	WT	Sputum	-	0.5	0.5	0.125
F610	25	6	3	3	5	3	5	2	7	3	4	6-3-3-5-3-5-2-7-3-4	KOREA	Non-CAPA	2022-02-09	WT	Sputum(Trap)	-	0.5	0.25	0.125
F613	69	7	4	3	5	3	4	2	2	2	3	7-4-3-5-3-4-2-2-2-3	KOREA	Non-CAPA	2022-02-17	WT	Sputum	-	0.5	0.5	0.125
F617	105	10	2	3	5	2	4	6	9	5	3	10-2-3-5-2-4-6-9-5-3	KOREA	CAPA	2022-02-23	WT	Bronchial washing fluid	-	0.5	0.5	0.125
F619	61	7	3	3	4	2	6	3	10	5	3	7-3-3-4-2-6-3-10-5-3	KOREA	Non-CAPA	2022-02-28	WT	Sputum	-			
F623	106	10	2	3	5	2	5	3	11	7	3	10-2-3-5-2-5-3-11-7-3	KOREA	Non-CAPA	2022-03-08	TR34	Bronchial washing fluid	L98H, S297T, F495I	**16**	**4**	**2**
F624	121	10	5	3	4	1	5	3	6	2	3	10-5-3-4-1-5-3-6-2-3	KOREA	Non-CAPA	2022-03-12	WT	Sputum	-	0.5	0.5	0.125
F628	2	3	5	3	5	3	6	2	6	4	3	3-5-3-5-3-6-2-6-4-3	KOREA	Non-CAPA	2022-03-21	WT	Pus	-	0.5	0.5	0.125
F630	82	8	2	3	5	3	4	3	6	4	3	8-2-3-5-3-4-3-6-4-3	KOREA	CAPA	2022-03-25	WT	Sputum	-	0.5	0.5	0.125
F631	112	10	2	5	6	1	4	2	11	3	2	10-2-5-6-1-4-2-11-3-2	KOREA	Non-CAPA	2022-03-29	WT	Sputum	-	0.5	0.5	0.125
F637	1	2	5	3	5	3	5	2	6	4	3	2-5-3-5-3-5-2-6-4-3	KOREA	CAPA	2022-04-18	WT	Sputum	-	0.5	0.5	0.125
F638	70	7	4	3	5	3	4	3	11	4	3	7-4-3-5-3-4-3-11-4-3	KOREA	Non-CAPA	2022-04-16	WT	Sputum	-	0.5	0.25	0.06
F647	11	6	14	3	7	1	5	2	14	2	3	6-14-3-7-1-5-2-14-2-3	KOREA	CAPA	2022-04-22	WT	Sputum	-	0.25	0.5	0.125
F649	129	11	3	3	5	2	4	2	12	1	3	11-3-3-5-2-4-2-12-1-3	KOREA	CAPA	2022-04-25	WT	Sputum(Trap)	-	0.5	1	0.125
F654	75	7	5	4	5	2	4	2	18	4	3	7-5-4-5-2-4-2-18-4-3	KOREA	Non-CAPA	2022-05-03	WT	others	-			
F660	42	6	5	3	5	3	5	5	10	3	3	6-5-3-5-3-5-5-10-3-3	KOREA	Non-CAPA	2022-05-21	WT	Sputum	-	0.5	1	0.25
F663	130	11	3	4	5	2	4	2	10	1	3	11-3-4-5-2-4-2-10-1-3	KOREA	Non-CAPA	2022-05-26	WT	sinus	-	0.25	0.5	0.125
F664	49	6	6	3	7	1	5	2	7	1	3	6-6-3-7-1-5-2-7-1-3	KOREA	CAPA	2022-06-15	WT	Sputum	-	0.25	0.25	0.125
F668	15	6	2	3	5	3	6	2	8	3	3	6-2-3-5-3-6-2-8-3-3	KOREA	Non-CAPA	2022-06-13	WT	Sputum	-	0.5	0.5	0.125
F669	109	10	2	3	7	3	4	2	8	3	3	10-2-3-7-3-4-2-8-3-3	KOREA	CAPA	2022-06-28	TR46	Eye discharge	Y121F, P216S, T289A, S363P, I364V, G448S	**32**	**64≤**	**32**
F671	131	11	5	3	5	3	4	3	14	3	3	11-5-3-5-3-4-3-14-3-3	KOREA	CAPA	2022-06-30	WT	Sputum(Trap)	-	0.125	0.125	0.125
F672	114	10	3	3	5	1	4	2	8	2	3	10-3-3-5-1-4-2-8-2-3	KOREA	CAPA	2022-06-20	WT	Sputum(Trap)	-	0.5	0.125	0.125
F673	43	6	5	3	5	3	6	2	6	3	3	6-5-3-5-3-6-2-6-3-3	KOREA	Non-CAPA	2022-06-17	WT	Bronchial washing fluid	-	0.5	0.25	0.125
F677	113	10	3	3	4	1	6	2	8	2	3	10-3-3-4-1-6-2-8-2-3	KOREA	Non-CAPA	2022-06-15	WT	Pus	-	0.25	0.5	0.125
